# Spatial O_2_ Profile in *Coix lacryma-jobi* and *Sorghum bicolor* along the Gas Diffusion Pathway under Waterlogging Conditions

**DOI:** 10.3390/plants13010003

**Published:** 2023-12-19

**Authors:** Shotaro Tamaru, Keita Goto, Jun-Ichi Sakagami

**Affiliations:** 1 The United Graduate School of Agricultural Sciences, Kagoshima University, Kagoshima City 890-0065, Japan; k5697087@kadai.jp (S.T.);; 2 Faculty of Agriculture, Kagoshima University, Kagoshima City 890-0065, Japan

**Keywords:** spatial oxygen profile, root anatomy, hypoxia, tissue oxygen, root cortex, root stele

## Abstract

While internal aeration in plants is critical for adaptation to waterlogging, there is a gap in understanding the differences in oxygen diffusion gradients from shoots to roots between hypoxia-tolerant and -sensitive species. This study aims to elucidate the differences in tissue oxygen concentration at various locations on the shoot and root between a hypoxia-tolerant species and a -sensitive species using a microneedle sensor that allows for spatial oxygen profiling. Job’s tears, a hypoxia-tolerant species, and sorghum, a hypoxia-susceptible species, were tested. Plants aged 10 days were acclimated to a hypoxic agar solution for 12 days. Oxygen was profiled near the root tip, root base, root shoot junction, stem, and leaf. An anatomical analysis was also performed on the roots used for the O_2_ profile. The oxygen partial pressure (pO_2_) values at the root base and tip of sorghum were significantly lower than that of the root of Job’s tears. At the base of the root of Job’s tears, pO_2_ rapidly decreased from the root cortex to the surface, indicating a function to inhibit oxygen leakage. No significant differences in pO_2_ between the species were identified in the shoot part. The root cortex to stele ratio was significantly higher from the root tip to the base in Job’s tears compared to sorghum. The pO_2_ gradient began to differ greatly at the root shoot junction and root base longitudinally, and between the cortex and stele radially, between Job’s tears and sorghum. Differences in the root oxygen retention capacity and the cortex to stele ratio are considered to be related to differences in pO_2_.

## 1. Introduction

Floods are categorized as submergence, partial submergence, waterlogging [1]. Submergence is a serious environmental stress that even threatens the production of rice, which is well adapted to wetland conditions [2]. Initially, almost all field crops struggle with the stress from partial submergence or even waterlogging, leading to restricted growth and yield. Global estimates indicate that approximately 10–12% of agricultural lands face waterlogging or severe soil drainage constraints [3]. In such conditions, the initial consequence is the depletion of oxygen in the soil, causing root hypoxia, which constrains nutrient absorption, root elongation, and maintenance [4]. Prolonged soil and root hypoxia disrupts the nutrient balance in plants, and reactive oxygen species can impact not only the roots but also the leaves [5]. The accumulation of reduced minerals [6] and soil phytotoxins [7], depending on soil type, can exacerbate this situation. Wetland plant species can endure such conditions due to their ability to continually supply oxygen to their roots [8]. Oxygen supplied to the roots not only sustains root respiration but also safeguards the roots from reducing conditions by oxidizing the root vicinity [9,10,11]. Comparative studies of plants in wetlands indicate that their ability to maintain respiration depends not on controlling the amount of respiration, but on supplying the necessary oxygen for respiration [12]. Therefore, while the stress induced by soil hypoxia is diversified by expanding spatiotemporal elements, the ability to provide oxygen to the roots is crucial from the outset.

The efficient supply of oxygen to the roots involves considering the characteristics of the above-ground parts as an oxygen supply source and the roots as a sink [8,13]. A meta-analysis integrating 129 studies on tissue oxygen concentrations in plants demonstrated longitudinal oxygen diffusion from the leaves to the roots [14]. Additionally, evidence from noninvasive methods using N-isotopes showed leaf-to-root gas diffusion in partially submerged rice plants [15]. Thus, the oxygen transport among the root, stem, and leaf organs is becoming clearer, but still only a limited number of these tissue oxygen dynamics have been elucidated in a single study. In particular, studies of oxygen dynamics in oxygen diffusion pathways under waterlogging conditions are limited, and tissue oxygen concentration profiles remain at the forefront. Concerning the significant contribution of the above-ground parts as an oxygen source, photosynthetically derived oxygen accumulates in the above-ground portions of submerged plants and serves as an oxygen source to the roots [16,17,18]. However, it remains unknown whether the oxygen transport properties of the leaves and stems contribute to interspecific differences in oxygen concentrations in waterlogged plants in which the above-ground parts are exposed to an atmosphere with a lower gas diffusion resistance than water. Although some studies suggest that differences in the above-ground parts’ porosity can affect gas diffusion to roots [19,20], no tissue oxygen data have been reported to discuss the extent of this effect under waterlogged conditions. Therefore, this study aims to elucidate the differences in the oxygen diffusion gradient from shoots to roots between hypoxia-tolerant and -sensitive species under waterlogging conditions. This investigation could offer valuable insights for enhancing plant internal aeration in the design of waterlogging-tolerant varieties. Micro-oxygen electrodes are a powerful tool to address this issue, combining high resolution, rapid response, and signal stability [21,22]. By ascertaining the distance at which the sensor is inserted by the manipulator, it is possible to measure the tissue oxygen concentration by matching the position of the tissue with the oxygen concentration [23,24,25].

In this study, we test Job’s tears (*Coix lacryma-jobi* var. *ma-yuen*; cv. Riogrande de sul) as a waterlogging-tolerant species and sorghum (*Sorghum bicolor*; cv. High-grain sorghum) as a waterlogging-sensitive species. Our endeavor is noteworthy as it introduces these two plant species, Job’s tears and sorghum, which have not been previously examined, to the existing collection of 112 species studied for tissue oxygen concentrations [14]. Tissue oxygen measurements have never been performed on these two species, but based on the stability of growth and transpiration under waterlogging in these two species [26,27], we hypothesize that tissue oxygen levels will be higher at least in the root tip of Job’s tears than in that of sorghum. It is worth highlighting that Job’s tears are considered the most closely related crop to sorghum [28]. The waterlogging-tolerant and humidity-induced disease-resistant traits observed in the Coix family, to which Job’s tears belong, are anticipated to contribute to the improvement in resistance breeding in maize and sorghum [29,30].

## 2. Results

### 2.1. The pO_2_ Profile in Root and Shoot

**Figure 1:**
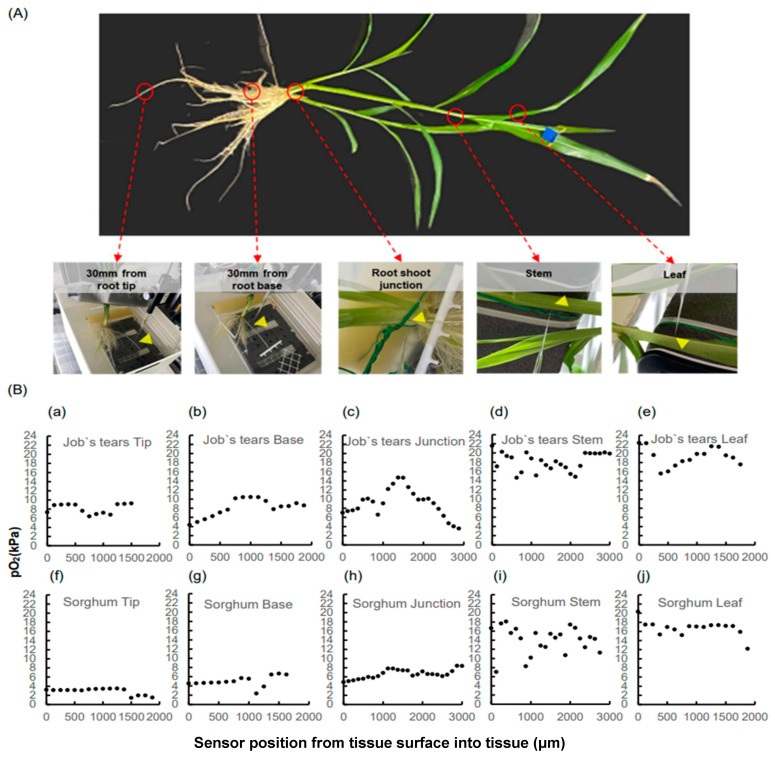
Example pictures of data collection locations (**A**) and data (**B**) illustrating pO_2_ profiles within the root structures of Job’s tears (**a**–**e**) and sorghum (**f**–**j**). The positive distances were measured at the root tip (**a**,**f**), root base (**b**,**g**), root shoot junction (**c**,**h**), stem (**d**,**i**), and leaf (**e**,**j**). The yellow arrows in the pictures indicate the points where the sensor was inserted. These points represent the median of the data collected >30 s. In this context, “tip” refers to precisely 30 mm from the root tip, “base” indicates precisely 30 mm from the root base, and “junction” means precisely within 10 mm from the root base on the root shoot junction. Profiles on the leaf were conducted in the youngest expanded leaf. Profiles on the stem were conducted near the auricle of the youngest expanded leaf.

The pO_2_ in the root was continuously measured in each sample until a steep O_2_ decrease was observed in the root stele. The highest peak of pO_2_ (9.3 kPa in the root tip of Job’s tears; 3.5 kPa in the root tip of sorghum) was found in the root cortex layer, while in the stele, pO_2_ (6.4 kPa in the root tip of Job’s tears; 1.5 kPa in the root tip of sorghum) decreased in each root (Figure 1a,b,f,g, respectively). The lowest pO_2_ values were collected as stele pO_2_ for statistical comparison. Additionally, Job’s tears showed a steep pO_2_ decrease across the root cortex to the root epidermis (41% lower in the epidermis relative to the cortex layer) at 30 mm from the root base (Figure 1b). Even at the root base, sorghum pO_2_ in the cortex and stele was lower than that of Job’s tears (62.4% lower in the cortex and 76.5% lower in the stele; Figure 1b,g, respectively). Therefore, we conducted a pO_2_ investigation of the above-ground parts using plants of the same age.

The results of the root shoot junction profile showed that the highest pO_2_ peaks (13.4 kPa in Job’s tears; 8.4 kPa in sorghum) were present in more internal tissues than on the tissue surface (7.0 kPa in Job’s tears and 4.9 kPa in Sorghum; Figure 1c,h, respectively). The highest peak of pO_2_ in the root shoot junction tended to be higher in Job’s tears than in sorghum (Figure 1c,h). However, the maximum pO_2_ in the root shoot junction of sorghum was 14.7 kPa, which was not inferior when compared to that of Job’s tears. The highest pO_2_ values of the shoot and leaves were clearly higher than those of the agar-submerged parts (Figure 1d,e,i,j, respectively). The gap in pO_2_ between the species was quite narrow on the leaf and stem exposed to the atmosphere (11.0% lower in the stem and 19% lower in the leaf) compared to the root.

The highest peak of pO_2_ in the root cortical layer and root shoot junction was used for statistical comparison as the pO_2_ in the intercellular space. To exclude direct atmospheric involvement, the highest peaks of pO_2_ of the leaf and stem inside the point where the pO_2_ decreased were used for statistical comparison as the intercellular space.

### 2.2. Statistical Comparison of the pO_2_ of Intercellular Space and Root Stele, and Gradient from the Cortex to the Epidermis

**Figure 2:**
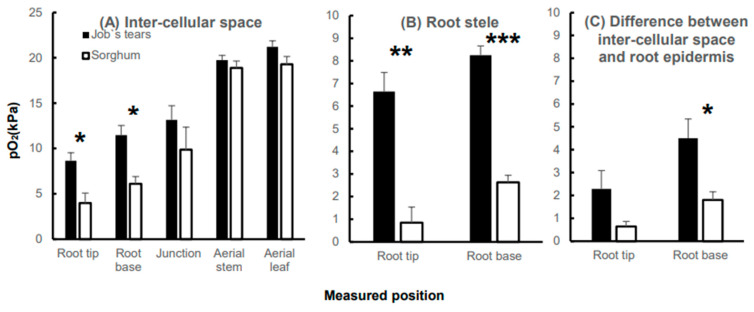
Statistical comparisons of the pO_2_ levels in different tissues of Job’s tears and sorghum. (**A**) depicts the pO_2_ in the intercellular space. (**B**) illustrates the pO_2_ in the root stele. (**C**) presents the pO_2_ difference between the intercellular space and the root epidermis. The bars represent the standard error (S.E.). Significant differences in pO_2_ between Job’s tears and sorghum were assessed using a Student’s *t*-test and denoted by asterisks: * 0.01 ≦ *p* < 0.05; ** 0.001 ≦ *p* < 0.01; and *** *p* < 0.001.

The pO_2_ levels in the intercellular space tended to decrease in the following order: from the root shoot junction to the root base, and root tip for both species compared to the stem and leaf exposed to the atmosphere (Figure 2A). The intercellular pO_2_ of sorghum was significantly lower than that of Job’s tears at the root base and near the tips, by 6.1 kPa and 4.0 kPa, respectively (Figure 2A). In terms of stele pO_2_, it was significantly lower, at 83%, at the root tip of sorghum and 68% lower at the root base, compared to the corresponding positions in Job’s tears (Figure 2C). To evaluate the ability to retain oxygen by preventing its diffusion from the root cortex to the epidermis, we calculated the pO_2_ difference between the intercellular space and the lowest pO_2_ around the root epidermis (within 250 μm). The statistical comparisons of these differences revealed that Job’s tears had a significantly higher pO_2_ difference near the root base than sorghum (Figure 2C).

### 2.3. Root Cross-Section Area and Cortex Area to Stele Area Ratio

**Figure 3:**
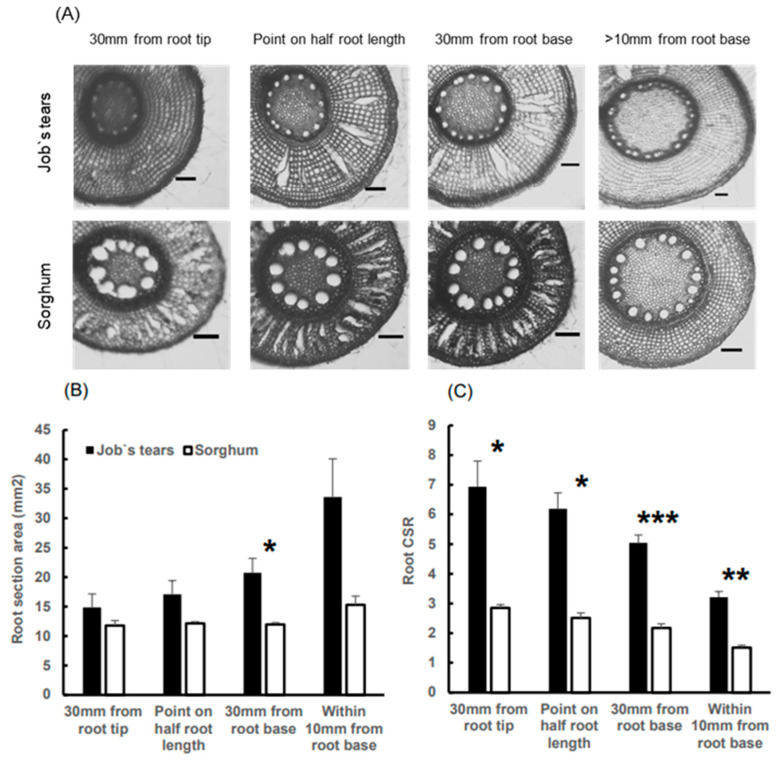
Examples of root cross-section (**A**) and statistical comparisons of the root cross-section area (**B**) and the root cortex area to the stele area ratio (**C**) in the O_2_-profiled roots of Job’s tears and sorghum. The black bars in A represent 500 μm. The bars in B and C indicate the standard error (S.E.). The significance between Job’s tears and sorghum was assessed using Welch’s *t*-test and denoted by asterisks: * 0.01 ≦ *p* < 0.05, ** 0.001 ≦ *p* < 0.01; and *** *p* < 0.001.

To explore the plant factors that influenc root pO_2_, we examined the pertinent characteristics through the dissection of the roots whose O_2_ level was profiled. The root aerenchyma was well identified in both species, especially from 30 mm from the root base to near the midpoint of the root, but was rarely identified within 10 mm of the base (Figure 3A). The root section area tended to be higher in Job’s tears than in sorghum within 10 mm from the root base (*p* = 0.09), with a significant difference observed at 30 mm from the root base (Figure 3B). Additionally, the root CSR was significantly higher in Job’s tears compared to sorghum at every sampled point (Figure 3C).

## 3. Discussion

In this study, we analyzed the tissue oxygen profiles from the leaves and stems to root tips for two plant species that differ in waterlogging tolerance. Initially, we explored the interspecific differences in pO_2_ values in the leaves and stems exposed to the atmosphere. When inserting the sensor into the stem, we observed points of extreme decrease in pO_2_ within the inner part of the stem (Figure 1d,j), possibly due to gas diffusion blocking the effect of the cuticle [31]. There was no significant difference between species in the value of the high peak, assumed to indicate a supply of oxygen to the surrounding tissues, and it was not significantly higher than the pO_2_ exposed to the atmosphere (Figure 2A). Contrary to observations in submerged plants [16,17,18], the high oxygen accumulation and its contribution to the roots were not observed under the waterlogged conditions in this study. As discussed by Sou et al., the oxygen produced by photosynthesis did not contribute to the aeration of adventitious roots in the water [32]. Additionally, the comparison of tissue oxygen concentrations in light and dark conditions revealed that the differences in the oxygen concentrations in the leaves, clear in submerged and partially submerged conditions, were no longer significant in waterlogging and drained conditions [33]. In summary, photosynthetically produced oxygen from leaves and stems in the atmosphere contributes harder to the roots under waterlogged conditions than under submergence. However, these studies, including our current study, were conducted indoors, and a more rigorous investigation of whether oxygen levels of tissues exposed to the atmosphere affect the submerged portion of the plant will need to be further tested in the field in the future to account for the effects of intense light and atmospheric conditions, as was conducted in field trials on submerged rice plants [34].

In this study, stem submergence in the agar solution mimicking waterlogged soil was 1–2 cm, yet the oxygen partial pressure decreased by 6.6 kPa (S.E. 1.9) in Job’s tears and 9.0 kPa (S.E. 1.7) in sorghum as the oxygen diffused from the stem into the root shoot junction (Figure 2A). Although interspecific differences at the root shoot junction were not significant, the differences were more pronounced than in the atmospheric stem and leaf (Figure 2A). The tested samples used in this study formed little aerenchyma within 10 mm of the root base, but interestingly, oxygen was transported at the root sites beyond that point. Although oxygen diffusion resistance is expected to be higher than in tissues with well-formed aerenchyma, oxygen transport through intercellular spaces is considered to occur even in such tissues [35]. Considering the pO_2_ of the aerial stem at 100%, 44% of the oxygen was diffused to the cortex near the root tip and 33% to the stele of Job’s tears, while in sorghum, only 21% of the oxygen reached the cortex near the root tip and a mere 4% the stele (Figure 2A,B). Insufficient oxygen reaching the root tips, the central metabolic site of the root, implies severe limitations to root activity [4,36]. Root stele hypoxia and anoxia severely restrict the loading of essential ions to the xylem [37,38,39,40]. When plants encounter hypoxia due to waterlogging, they adapt by switching metabolic systems early in the stress period, but morphological changes are essential for adaptation to long-term stress [41]. In susceptible field crops, like wheat, roots that develop after encountering hypoxic stress are more adapted to hypoxia than roots that develop before the stress encounter [42]. In our study, sorghum exposed to 12 days of hypoxia exhibited a fundamental difference in oxygen transportation compared to Job’s tears. This underscores the need to address weaknesses in plant tissues exhibiting differences in oxygen concentration in field crops such as sorghum. Consequently, we will continue our study of root properties affecting oxygen diffusion longitudinally and radially.

The pO_2_ difference between the two species increased significantly as the oxygen diffused from the root shoot junction to the root base (Figure 2A). Oxygen retainability at the root base of Job’s tears was markedly higher than that of sorghum (Figure 2C), suggesting the presence of a ROL barrier-like function in the roots of Job’s tears. A similar pattern of tissue oxygen concentrations with a decrease in epidermal oxygen concentrations in the cortical layer has been reported in *Glyceria maxima*, which suppresses oxygen leakage from the roots [43]. The ROL barrier serves to prevent oxygen loss from the root base, enabling extended oxygen transport [9]. The ROL barrier has never been tested in Job’s tears. The ROL barrier has been termed the outer apoplastic barrier in a recent study and has multiple functions [44]. Future studies should examine the extent to which Job’s tears has any of these functions. Moreover, the advantage of possessing thicker and higher CSR roots for oxygen transport, as noted by Yamauchi et al. [40], is consistent with the characteristics of the roots of Job’s tear in this study (Figure 3B,C). The differences in longitudinal oxygen diffusion, influenced by high aeration and oxygen retention, contribute to variations in pO_2_ in the intercellular space at the root tips. Additionally, the formation of a larger cortical layer is crucial for facilitating sufficient oxygen diffusion to the stele [45]. The CSR of Job’s tears exceeded that of sorghum from the root base to the root tips, resulting in a radial difference in oxygen penetration into the stele. The chromosomal region controlling the inducible ROL barrier has already been identified [46], and there is a possibility of further QTL studies into the constitutive ROL barrier since the discovery of the wild rice that forms it [47]. In contrast, CSR regulation is a more recently discovered phenomenon than the ROL barrier, and its genetic domain has not yet been fully elucidated [48,49].

Utilizing the spatial measurements of tissue oxygen partial pressure, this study unveiled where differences in oxygen permeability occur in closely related species, sorghum and Job’s tears, under hypoxic agar-mimicked waterlogged soil conditions. However, this does not imply the absence of tolerant species with clearer differences in oxygen transport capacity at the root shoot junction. For instance, compared to previous studies, Nicaragua teosinte (14.0 kPa) exhibited a higher oxygen partial pressure at the root base [50] than the root base of Job’s tears (11.5 kPa) in this study. It is conceivable that the above-ground aerating capacity is similar or even higher in such plants. In addition, although the present study compared stress-acclimated plants, waterlogging-sensitive crops do not a have constitutive aerenchyma, a trait present in adapted species [51,52], and therefore, tissue oxygen differences may be even greater at the early stages of stress. How hypoxia is sensed and morphological adaptation occurs are still matters of debate for hypoxic-sensitive plants [53], and therefore, a follow-up comparison from the early stages of stress to stress acclimation should be investigated in future studies. At present, certain traits beneficial for adaptation to waterlogged environments are associated with trade-offs in adaptation to drought environments [54,55]. Therefore, traits with low adaptive costs that provide sufficient oxygen permeability for adaptation to target flooding environments need to be incorporated into field crops. The proper evaluation of such characteristics requires future research linking oxygen levels to traits in various crops.

## 4. Materials and Methods

### 4.1. Plant Material and Sample Preparation

Seeds from each species were sterilized using a 0.5% sodium hypochlorite solution for 30 min, followed by rinsing with distilled water. The sterilized seeds were then placed in Petri dishes with distilled water and incubated for three days at 31 °C. The germinated seeds were subsequently transferred to a 10 L tank filled with a hydroponic solution (Hyponica, Kyowa, Osaka, Japan; contents were as shown in Tada et al. [56]; pH was adjusted to 5.5–5.7 after dilution) diluted 500 times and floated for seven days at 27 °C. Light and dark cycles were set at 12 h each, with a photosynthetic active radiation of >400 μmol m^−2^ s^−1^ on the leaves during the daytime. The light source was a white light-emitting diode (SMD-50W, Gentos, Tokyo, Japan). Following this, the plants underwent a 12-day hypoxic acclimation process. Each of the three plants was grown in a 0.1% nutrient agar solution that was flushed with N_2_ gas until the dissolved oxygen concentration was lower than 1.0 mg/L. This 0.1% agar solution is a useful material to mimic the gas convection process under waterlogged soils [57]. The nutrient and atmospheric conditions were the same as those after germination. The 0.1% nutrient agar solution was renewed once on the 7th day after treatment. Plants on the 13th day of hypoxia acclimation were used for the subsequent O_2_ measurement.

### 4.2. Micro-O_2_ Profiles in Root and Shoot Tissues

To profile the oxygen status in the root tissue (30 mm from the root tip and 30 mm from the root base), a microsensor with a 25 μm tip diameter (OX25, Unisense A/S, Aarhus, Denmark) was utilized. For measurements in the shoot tissue (root shoot junction, stem, and leaf exposed to the atmosphere), a microsensor with a 100 μm tip diameter (OX100, Unisense A/S, Denmark) was employed. Each sensor was connected to a picoampere meter (UniAmp Single Channel; Unisense A/S, Denmark) and polarized overnight one day prior to each measurement. An intact plant was secured in a 2 L tank filled with 0.1% agar solution. For each measurement, the stems were submerged in the agar solution at 1 to 2 cm. A white light-emitting diode (SMD-50W, Gentos, Japan), serving as the light source, was installed for each measurement, providing photosynthetic active radiation to the leaf tip (500 μmol m^−2^ s^−1^), leaf base (200 to 210 μmol m^−2^ s^−1^), and stem (120 to 150 μmol m^−2^ s^−1^). The sensor, calibrated following the method by Jiménez et al. [58], was mounted on a micromanipulator (MP-2, Narishige Group, Tokyo, Japan). For the submerged parts (30 mm from root tip, 30 mm from root base, and root shoot junction), the sensor was carefully inserted into the agar solution, with the sensor tip monitored by an endoscope until it reached the tissue surface. For the aerial parts (stem and leaf), the sensor was carefully positioned to reach the tissue surface, guided by visual observation. Measurements were taken at intervals of 125 μm for over 30 s, and sensor signals were collected using data acquisition software (SENSORTRACE SUITE v.3.4.400; Unisense A/S, Denmark). The dissolved oxygen concentration in the agar solution was measured before the measurements, and if it exceeded 1.0 mg/L, N_2_ gas was flushed out of the plant to maintain the concentration below 1.0 mg/L. Measurements on the root were taken at 27.5 °C, while those on the shoot were taken at 26.0 °C. Since the units of the oxygen concentration values obtained were in μmol/L, they were converted to kPa using the following equation.

$$pO_{2}=100\frac{10^{-6}[O_{2}]}{K(T)}$$

where pO_2_ is the partial oxygen pressure. [O_2_] is the measured value (μmol/L) using the micro-oxygen sensor. K(T) is Henry’s constant at temperature T. In this study, 0.00128 mol/(kg·bar) (27.5 °C) and 0.00123 mol/(kg·bar) (26.0 °C) were used as the Henry’s constant values to calculate the partial pressure of the oxygen dissolved in water. To convert bar to kPa, we multiplied the values by 100.

### 4.3. Root Dissection and Measurements of Root Anatomical Traits

Following the O_2_ measurements, the roots that were measured were immediately cut from the shoot and stored at 4 °C. Cross-sections of the roots were obtained at 30 mm from the tip, at the halfway point between the two O_2_ measurements, 30 mm from the root base, and within 10 mm from the root base. These sections were prepared manually using a razor blade and were immediately placed on glass slides. Each section was photographed using a microscope (MP38T, As one, Osaka, Japan), equipped with a microscope camera (PCM500, As one, Japan). The outlines of the tissues (the whole area of the cross-section, the internal area of the epidermis, and the stele area) in the cross-sectional images were traced by freehand selection, and their areas were quantified using the ImageJ software (v.1.53e; National Institutes of Health, Bethesda, MD, USA). The root cross-section cortex area to stele area ratio (CSR) was calculated by dividing the cortex area (calculated by subtracting the stele area from the internal area of the epidermis) by the stele area.

### 4.4. Statistical Analysis

The Shapiro–Wilk normality test, F-test, Student’s *t*-test, and Welch’s *t*-test were conducted using the EZR software (v.1.61), which was programmed by Kanda [59] and is based on R software (v.4.2.2). Some datasets of the root cross-section area and root CSR of position between Job’s tears and sorghum included unequal variance. Therefore, Welch’s *t*-test was used for these parameters instead of the Student’s *t*-test.

## 5. Conclusions

From the longitudinal and radial comparisons of oxygen partial pressure from the leaves to the root tips, notable differences in the pO_2_ gradient emerged, particularly at the root shoot junction and root base longitudinally, as well as in the root cortex and root stele radially between Job’s tears (cv. Riogrande de sul) and sorghum (cv. High-grain sorghum). The oxygen retainability of the root and thicker and higher CSR roots contributed to longitudinal oxygen diffusion in Job’s tears. The high CSR allowed for the higher radial oxygen diffusion to the stele of Job’s tears.

## Data Availability

Data will be made available upon request.
